# Stability of Octadecyltrimethoxysilane-Based Coatings on Aluminum Alloy Surface

**DOI:** 10.3390/ma15051804

**Published:** 2022-02-28

**Authors:** Alexey Y. Zhizhchenko, Anastasiia V. Shabalina, Ali A. Aljulaih, Stanislav O. Gurbatov, Aleksandr A. Kuchmizhak, Satoru Iwamori, Sergei A. Kulinich

**Affiliations:** 1 Far Eastern Federal University, Vladivostok 690091, Russia; g89leksig@mail.ru (A.Y.Z.); gurbatov_slava@mail.ru (S.O.G.); alex.iacp.dvo@mail.ru (A.A.K.); 2 Institute of Automation and Control Processes of FEB RAS, 5 Radio St., Vladivostok 690041, Russia; 3 Siberian Physical-Technical Institute, National Research Tomsk State University, Tomsk 634050, Russia; shabalinaav@gmail.com; 4 Department of Mechanical Engineering, Tokai University, Hiratsuka, Kanagawa 259-1292, Japan; ali.aljulaih@gmail.com (A.A.A.); iwamori@tokai-u.jp (S.I.); 5 Division of Physical Science & Engineering, King Abdullah University of Science and Technology (KAUST), Thuwal 23955-6900, Saudi Arabia

**Keywords:** octadecyltrimethoxysilane, wettability, aluminum alloy, SEM, surface corrosion

## Abstract

Long-term stability in contact with water of organosilane layers formed by octadecyltrimethoxysilane (ODTMS) on polished aluminum alloy (AA2024) through dip-coating was studied by combining SEM, water contact angle measurements, and X-ray photoelectron spectroscopy. Similar organosilane layers were formed on AA2024 coated with permanganate conversion coating, 1,2-bis(triethoxysilyl)ethane (BTSE) and hydrated SiO_x_ as under-layers, after which their long-term durability was also tested. During immersion in water for about one month, all the samples exhibited a decrease in hydrophobicity, implying the prepared organosilane layer was not stable over time, gradually hydrolyzing and letting water interact with the underlying layer. In parallel, SEM images of one-layer samples taken after immersion showed clear signs of local electrochemical corrosion, while XPS analysis confirmed a loss of silicon from the surface layer. The highest stability over time was demonstrated by a one-layer sample prepared in an ethanol/water bath for 5 min and by a similar ODTMS layer prepared on hydrated MnO_x_ as an under-layer.

## 1. Introduction

Hydrophobic and superhydrophobic surfaces have been a subject of active research for several decades as they present interest for both fundamental science and industry [1,2,3,4,5,6,7,8,9,10,11,12,13,14,15,16,17]. The water-repellent properties of materials are known to be either provided by their intrinsically low surface energy or by depositing a thin top-layer with low energy. Increased surface roughness can render material superhydrophobicity, while smooth surfaces are known to demonstrate water contact angle (CA) only as high as ~120° [3,4,5,6,7,8,9,10,12,14,15,18,19,20].

Self-assembled ultrathin monolayers of organosilanes, which are one of the most commonly used low-surface energy coatings (often referred to as ‘hydrophobizers’), have been applied on metal, oxide, ceramic and Si substrates [1,8,14,16,17,18,19,20,21,22,23,24,25,26,27,28,29,30,31,32]. For several decades, various organosilanes, including fluorinated alkylsilanes (FASs), have been used as hydrophobizing agents deposited either via immersion in liquid bath [1,8,14,21,26,27,32,33,34,35] or from gas phase [13,27]. Apart from water repellency, which is normally expected in case of alkylsilanes, organosilane layers were also reported as part of anti-ice (or ice-phobic) surfaces [4,8,14,16,17,36,37], as well as part of anticorrosive coatings [21,22,23,27,32,33,38,39,40]. 

Various organosilane-based coatings were reported to be effective as anticorrosive layers on metal surface [21,22,23,24,25,26,27,40], including steel [25,33,38], copper [24], magnesium [21,26], and aluminum alloys [22,23,26,27,32,40]. To some extent, such coatings are even considered as potential alternatives to conversion coatings, which are still widely used for metal protection [26,27,40,41,42,43,44]. Finally, most recently, certain hydrophobic silanes, chiefly alkylsilanes and fluoroalkylsilanes, have been proposed and tested as materials with reduced wet-snow and/or ice accumulation on various materials and surfaces [3,4,8,14,16,17,36,37]. Therefore, they need to meet basic requirements for outdoor applications, such as thermal and chemical stability, UV and rain/snow resistance, and durability, as well as resistance against long-term contact with water. At the same time, no systematic investigations of long-term stability of silane-based coatings have been carried out so far.

To date, formation mechanisms for various silanes on diverse substrates (e.g., Si, silica, mica, and alumina) have been studied extensively [18,19,20,23,34,45,46,47]. Thermal stability of various alkylsiloxane self-assembled monolayers on silica or SBA-15 surfaces was also investigated both in air and in vacuum [28,29,30,31]. For instance, Kulkarni et al. assessed thermal stability of octadecyltrichlorosilane (OTS) monolayers on both Si substrate (planar surface) and silica spheres (curved surface) using results of various surface-sensitive spectroscopic techniques [30]. Densely packed OTS monolayers on a flat Si surface were found to be thermally stable up to 250 °C, while significant enhancement in thermal stability was observed for the case of OTS (up to 350 °C) on freshly prepared spherical SiO_2_ surfaces [30]. Thermogravimetric analysis showed the stability of OTS layers on a curved surface up to 350 °C in air and complete decomposition of monolayer that took place around 600 °C [30]. At the same time, OTS layers on a planar surface were found to be stable up to 250 °C, and their complete decomposition took place around 400 °C. The IR results showed that the monolayer decomposition took place through the Si–C and C–C bonds. The thermal behavior of the monolayer did not depend on the Si–O–Si linkage between the adjacent chains, and it remained intact up to 830 °C [30]. Independently, Mirji et al. used thermo-gravimetric analysis to reveal that OTS layers adsorbed on mesoporous SiO_2_ were stable up to 230 °C and decomposed between 230 and 400 °C [31].

Information on long-term stability of various hydrophobic materials immersed in water is rather scarce and not systematic [2,5,6,7,8,11,15,35,37,48]. Typically, some drop in CA values was observed after prolonged contact with water for as long as hundreds of hours, with hydrolysis proposed as the main possible explanation for observed results [5,7]. Similar experiments on silane-based coatings (as one of commonly used hydrophobizers) were not carried out systematically. Self-assembled monolayer of OTS prepared on Si substrate was assessed by CA measurements and XPS as it was immersed in water, exhibiting no appreciable changes after ~24 h of exposure to water [11]. Also, no change in CA was observed for FAS-coated aluminum whose durability was tested under running water drops [35]. At the same time, superhydrophobic aluminum coated with FAS demonstrated reduction in CA after tens of hours of contact with water [5]. Similar findings were reported by Yang et al. who tested superhydrophobic surfaces based on rough WO_x_ coated glass which was then hydrophobized by FAS [7]. Finally, OTS coated superhydrophobic titanium surfaces were found to exhibit reduced CA after immersion in liquids with pH of 1 and 13 for as long as several days [2]. 

This work aimed at systematically studying the hydrophobic properties of polished (flat) aluminum surfaces coated with an octadecyltrimethoxysilane (ODTMS) layer as they were immersed in water. Both surfaces coated with ODTMS (as one layer) and with an under-layer and ODTMS as a top-layer were tested. We used the following coatings as under-layers: (i) permanganate conversion coating, (ii) organosilane layer based on BTSE, and (iii) hydrated SiO_x_ layer prepared through hydrolysis of TEOS. For comparison, another one-layer ODTMS sample was subjected to the ultrasonic rinse test, which simulated a long-term water immersion test over a short period of time. All the samples showed gradual decay of their water-repellent properties, which should be related to gradual hydrolysis of ODTMS molecules grafted to the surface via their Si-O group.

## 2. Experimental Part

ODTMS (98% pure), bis-1,2-(triethoxysilyl)ethan (BTSE, 99% pure) and tetraethoxysilane (TEOS, 99% pure) reagents were all purchased from Sigma-Aldrich and used as supplied. Deionized water produced by a Milli-Q system (with the resistivity of 18.2 MΩ·cm) was used in all experiments. Aluminum alloy plates (AA2024, 2.5 × 2.5 cm^2^ in size) were used as substrates for all samples. This alloy is known to be widely used in various fields, including aviation, with its main additives being Cu, Mg, Mn, and Fe and the main intermetallic particles precipitated in its Al matrix being Al-Cu-Fe-Mn and Al-Cu-Mg types [41,42,43,44,49,50]. For more details on its additives, please see Table S1 in Supplementary Materials. Both one-layer and two-layer coatings were prepared, the former samples (see samples 1–3, Table 1) having only an ODTMS layer and the latter samples (samples 4–6, Table 1) having an under-layer and a top-layer of ODTMS. Table 1 presents a more detailed description of the samples tested in this work. 

**Figure 1:**
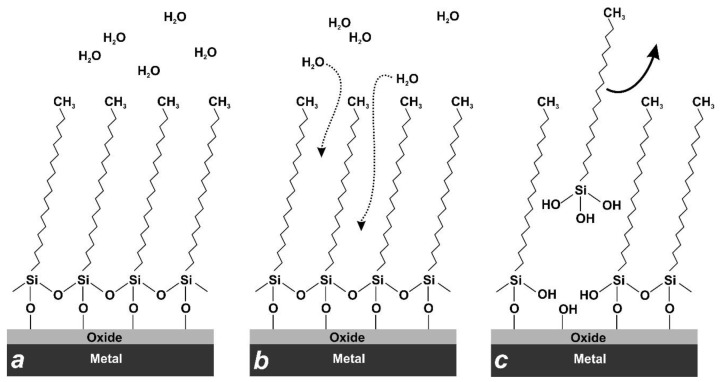
Schematic presentation of self-assembled one-layer ODTMS coating formed on metal oxide substrate. (**a**) Dense ODTMS layer with hydrophobic methyl-groups that repel water. (**b**) Water molecules gradually penetrating inside the layer over time to attack and hydrolyze the Si-O-Si and Si-O-Al bonds. (**c**) Loose alkylsilanol molecules are released from the coating as a result of hydrolysis, leaving unprotected sites.

Sample 1 was a plate of polished AA2024 coated with ODTMS. It was first degreased via sonication in methanol (10 min), then mirror-polished (subsequently with 1.0 and 0.5-µm sized alumina slurry), after which it was sonicated in water for 5 min, then ultrasonically cleaned in methanol and ethanol (10 min each). Finally, it was dried in air for 1 h, which allowed formation of a thin oxide layer, prior to further immersion into coating bath [17,34]. The bath used contained 1% of ODTMS in water/ethanol (1:9 *v*/*v*) solution. After immersion for 30 s, the sample was removed from the bath, rinsed and sonicated in ethanol, blow-dried with nitrogen flow, and finally heat-treated overnight at ∼50 °C in air [17,34]. Sample 2 was prepared using the same protocol, except that immersion time was 5 min to test the effect of bath treatment time. Similarly, sample 3 followed the preparation protocol used for sample 2 but methanol was used instead of ethanol to verify the effect of solvent (see Table 1). Figure 1a presents schematically one-layer ODTMS coatings formed on samples 1–3. 

Aiming at increasing the density of surface OH groups (and thus at possible preparation of denser ODTMS layers on such surfaces), we also prepared three samples with various hydrated under-layers based on different materials terminated with hydroxyl groups. Such under-layers with different chemistries were first prepared, after which a top-layer of ODTMS was prepared on fresh surface following the same protocol as described above for sample 2. 

Sample 4 was first coated with a permanganate conversion coating (PCC), which is based on hydrated MnO_2_ and known to protect well against corrosion [41,42,43]. In addition, such a hydrated PCC layer was expected to provide a larger surface density of OH groups to provide a denser ODTMS layer grafted on the surface. Upon polishing and ultrasonic cleaning, the sample was first immersed in a KMnO_4_-borax bath at 25 °C, the coating recipe being described in greater detail elsewhere [41,43]. After 2.5 h, it was removed from the bath, rinsed with deionized water, blow-dried with nitrogen and then immersed in the ODTMS bath for 5 min (similar to sample 2). 

Sample 5 was first coated with a BTSE layer, following the recipe previously presented elsewhere [39,51]. To provide a maximum amount of silanol groups (and consequently, the highest surface density of hydroxyl groups), the pH was kept in the 4.5–5.0 range [39]. A 4.0% BTSE bath (*v*/*v*) was used, with dip-coating time set as 100 s, to provide a layer thickness on the order of 100 nm [39,51], and the sample was finally dried in air prior to further treatment in the ODTMS bath. 

For sample 6, its under-layer was prepared from TEOS and was based on hydrated silica, following the protocol previously described by others [33]. Mirror polished AA2024 substrate was sonicated in methanol, after which a hydrated SiO_x_ layer (with a high density of surface hydroxyl groups [33]) was prepared by successive immersion in neat TEOS (each time for 15 s) followed by immersion in deionized (DI) water for 2 min, followed by drying in N_2_ gas flow. Both immersion in TEOS and water were repeated 5 times, after which the sample was kept in water for 1 h and then well rinsed with DI water. Finally, the sample was blown with nitrogen and immersed in the ODTMS bath for grafting with an organosilane top-layer [33]. 

Water-repellent characteristics of the samples (water contact angle, CA, and contact angle hysteresis, CAH) were evaluated on a commercial contact-angle goniometer (DSA100, from Krüss, Hamburg, Germany) following standard procedures. CA values were measured using water droplets of 5 μL in volume gently placed on the surface, after which their shape was evaluated by using the goniometer optics and software. CAH was evaluated as the difference between the advancing and receding CAs which were observed when water was added to or sucked off from the droplet placed on the substrate. Both CA and CAH values reported here were the average of at least five measurements on various surface locations of each sample. Scanning electron microscopy (SEM, S4800 from Hitachi, Tokyo, Japan) was used to take surface images of the samples. To reduce sample surface charging, low acceleration voltage of 5 kV was applied during SEM analyses. X-ray photoelectron spectroscopy (XPS) analysis was performed in a PHI-1600 spectrometer (Physical Electronic Industries, Chanhassen, MN, USA). All binding energies were corrected for charge shifting by referencing to the adventitious carbon C1s line at 285 eV.

The ultrasonic rinse test was previously proved to be a simple but useful approach to estimate strength of chemisorbed organosilane molecule—metal oxide bond, as well as stability of the organosilane layer [51,52]. Within this approach, we immersed sample 2 in water and subjected to ultrasonic treatment for 1 h. This treatment was used to simulate accelerated interactions between the surface-grafted ODTMS molecules and water.

## 3. Results and Discussion

### 3.1. Surface Morphology and Water Repellency of As-Prepared Samples

**Figure 2:**
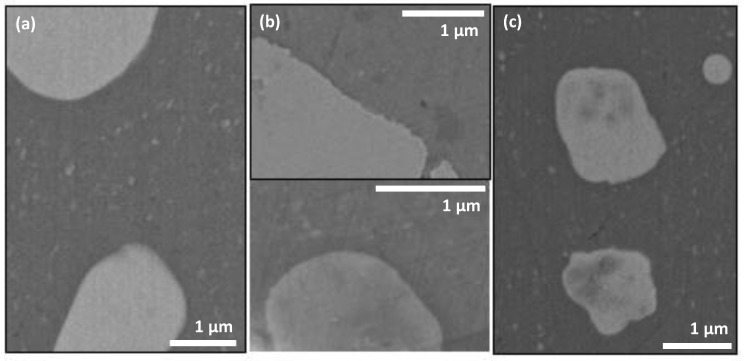
SEM images of as-prepared one-layer samples 1 (**a**), 2 (**b**), and 3 (**c**). All the surfaces are coated with one layer ODTMS coating. No traces related to electrochemical corrosion are seen on the surfaces. In panel (**b**), interface between two intermetallic particles (Al-Cu-Fe-Mn and Al-Cu-Mg types, top and bottom, respectively) and Al matrix are shown, both being corrosion-free.

**Figure 3:**
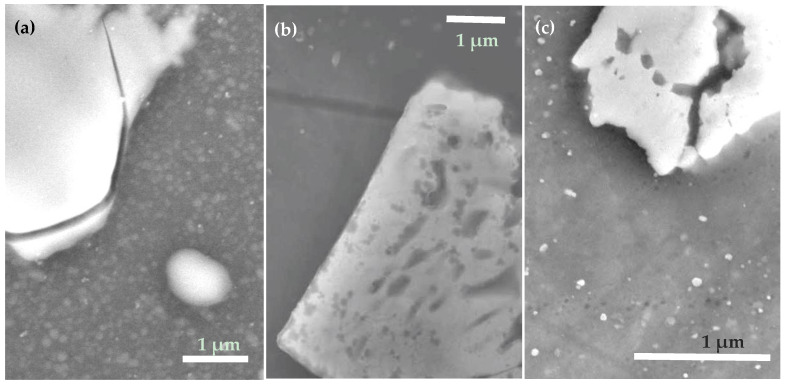
Surface SEM images of two-layer coatings: samples 4 (**a**), 5 (**b**), and 6 (**c**). All the surfaces are coated with ODTMS top-layer, with no traces of surface corrosion seen.

Surface images of as-prepared ODTMS-coated samples is presented in Figure 2 (samples 1–3) and Figure 3 (samples 4–6). Figure 2 shows that all one-layer samples are smooth and demonstrate no noticeable signs of surface corrosion, which is normally associated with surface trenches and re-deposits consisting of metal oxides and hydroxides observed mainly around and in the vicinity of second-phase particles [8,22,26,42,44,53]. Such trenches appear on the surface of AA2024 in the presence of electrolytes or water as a result of electrochemical couples that form between the aluminum matrix and second-phase particles embedded in it [42,49,50]. When the AA2024 surface is not well protected from electrolytes, the difference in chemical potential between the matrix and second-phase intermetallics typically causes gradual dissolution of the matrix adjacent to a cathodic particle [49,50,53]. Since all intermetallic particles are well seen in Figure 2 to exhibit very smooth boundary with their surrounding alloy matrix, one can conclude that all the samples coated with one layer were well protected with ODTMS to resist air humidity. It is clearly seen in Figure 2 that both smaller intermetallic particles of the Al-Cu-Mg type (panels (a), (c), and bottom of panel (b)) and bigger irregular-shaped Al-Cu-Fe-Mn particles (panel (b), top) showed no signs of surface corrosion. 

The surface morphology images of samples 1–3 presented in Figure 2a–c imply that the surface smoothness of all the three one-layer samples was very similar. This is also confirmed by their roughness parameters evaluated by profiler (see Table S2). Meanwhile, the wetting properties of the samples were somewhat different (see initial CA values for samples 1–3 in Table 1), which is explained by difference in their ODTMS layers. As shown below in Section 3.2, sample 2 demonstrated the best stability over time, implying that its preparation conditions were best optimized. That is why, hereafter, we focused mainly on sample 2 and prepared all the two-layer samples using its conditions for their top-layer.

Analysis of data presented in Figure 2 and Table 1, as well as below in Section 3.2, permits to conclude that in general our results are consistent with those previously reported by Thomsen et al., who reported that ODTMS formed a self-assembled layer on Al surface as quickly as after just ~1 min [34]. This explains why sample 1, which was prepared via a short immersion in ODTMS bath for only 30 s, demonstrated a slightly lower CA (see Table 1) and turned out to be less stable over time. It is reasonable to assume that its ODTMS layer was not assembled well enough and formed an insufficiently dense coating. 

Surface images of two-layer samples are presented in Figure 3, where panels (a–c) represent surface morphology of samples 4, 5, and 6, respectively. This time, because the under-layers were much thicker than a single layer of ODTMS, the surfaces in Figure 3 are seen to be much rougher than those in Figure 2. In addition, the intermetallic particles on the surface of samples 4–6, as well as their boundaries with the aluminum matrix, are seen to be well coated. Therefore, no trenches at the boundary are expected as the surfaces are coated with under-layers that are thick enough to block surface corrosion under the conditions used in this study. 

In agreement with Figure 2 and Figure 3, the CA values of as-prepared samples are seen in Table 1 to present generally flat hydrophobic surfaces. The difference in CA values observed for the samples is explained by: (i) some difference in assembly order/disorder of ODTMS layer formed on flat surface (governed by immersion time and solvent, to name the main parameters); (ii) surface chemistry of the substrate on which ODTMS layer is formed; and (iii) surface roughness. That is why it is reasonable to observe somewhat higher initial CA values on the surface of two-layer samples 4–6 as those samples are seen in Figure 3 to have somewhat higher surface roughness. 

### 3.2. Long-Term Water Repellency of Samples with One-Layer Coating

**Figure 4:**
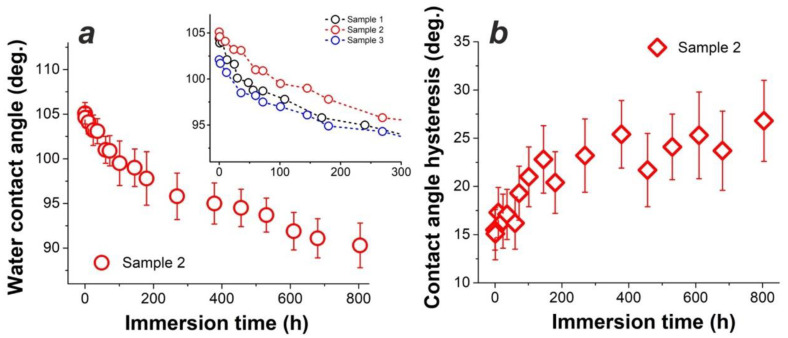
Wettability of sample 2 immersed in DI water for a long time. (**a**) Water CA values measured during immersion for more than 800 h. (**b**) Water CAH values measured for the same sample over time. Inset in panel (**a**) compares samples 1, 2, and 3 as their CA values are plotted vs. immersion time.

Figure 4a demonstrates how CA values of one-layer samples 1, 2, and 3 changed over time as the samples were immersed in DI water. Panel (a) shows that sample 2 was gradually losing its hydrophobicity as its CA dropped from ~105° to ~90° after as long as 800 h of immersion, while the inset in Figure 4a compares water-repellent behavior of samples 2 (red circles), 1 (black circles), and 3 (blue circles). It is seen that sample 2 exhibited the highest water-repellent properties, implying that its ODTMS layer was probably best assembled of all the three samples. This justifies why in our experiments with two-layer samples the second layer was deposited following the protocol used for sample 2. 

**Figure 5:**
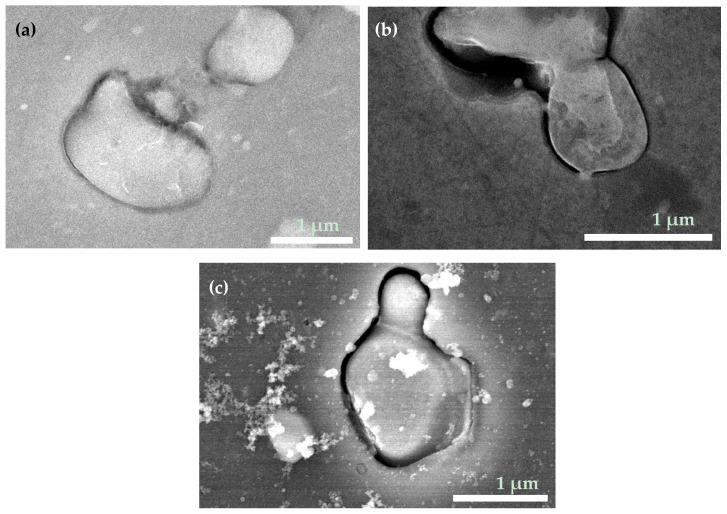
Surface SEM images of sample 2 after immersion into DI water for 24 h (**a**,**b**) and after sonication in DI for 60 min (**c**). Trenches surrounding surface intermetallic particles are seen, being a sign of electrochemical corrosion [8,41,42,43,49,50].

It is clearly seen in Figure 4b that in parallel, the values of CAH measured on sample 2 gradually increased, implying that its surface tended to become more inhomogeneous over time. To get more information, surface SEM images for sample 2 kept in water for 24 h are given in Figure 5a,b. Comparison of the intermetallic particles (both are of Al-Cu-Mg type) seen in panels (a) and (b) with their counterpart in Figure 2b (bottom) permits to conclude that electrochemical dissolution took place in the vicinity of such intermetallic particles during immersion in water. This suggests that immersion in DI water led to a gradual degradation of the ODTMS layer, after which the poorly protected patches of AA2024 surface were subjected to surface corrosion well described in the literature [49,50]. As electrochemical dissolution of intermetallic particles and their surrounding Al matrix proceeded over time, the sample surface turned more and more chemically inhomogeneous, which manifests as a gradual increase in CAH as shown in Figure 4b. 

Another possible explanation for the results presented in Figure 4 and Figure 5 could be a relatively poor density of the ODTMS layer formed on AA2024 (sample 2), which would allow water molecules to penetrate through the coating and initiate electrochemical processes on the substrate surface. In this scenario, the results observed in Figure 4 could be explained without involving the degradation of ODTMS-substrate bonds. To verify this possibility, below, we discuss the wetting behavior of two-layer samples immersed in water for similar periods of time.

### 3.3. Water Repellency of Two-Layer Samples over Time

**Figure 6:**
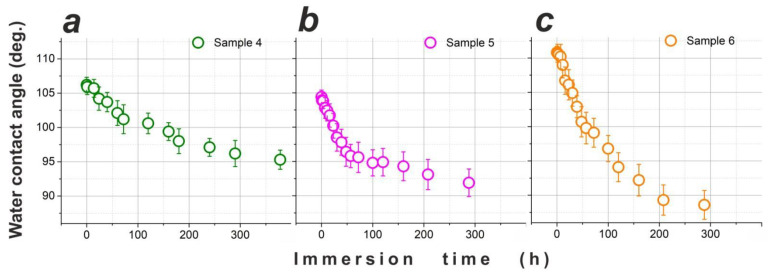
Wettability of samples 4 (**a**), 5 (**b**), and 6 (**c**) immersed in DI water for long time. Water CA values are shown as they evolved for as long as ~300–400 h of immersion.

**Figure 7:**
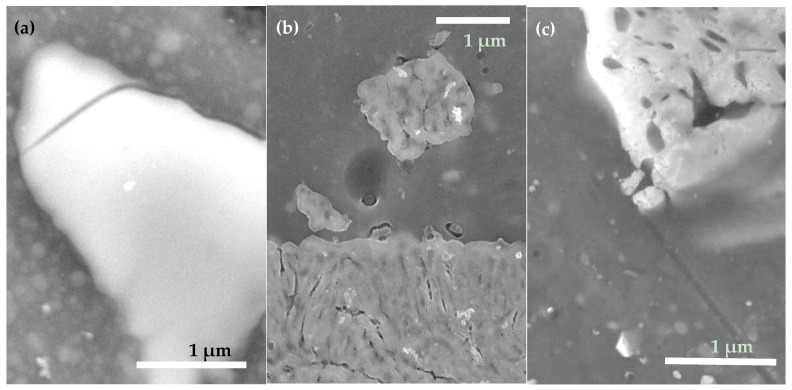
Surface SEM images of samples 4 (**a**), 5 (**b**), and 6 (**c**) after immersion in DI water for 72 h.

Similar to samples 1–3, samples 4, 5, and 6 were also tested in terms of their long-term stability in water. Their CA values measured over time are presented in Figure 6, and their surface images taken after immersion for 72 h are presented in Figure 7. Since the under-layers of these samples had quite different surface chemistry and morphology (see Figure 3), different surface density of ODTMS molecules that formed their top-layer was expected. Therefore, improved stability of the ODTMS monolayer could be expected in such a case.

However, as seen in Figure 6, all three of the two-layer samples (with different under-layers and ODTMS top-layer) demonstrated behavior quite similar to that previously observed for samples 1–3. All the samples are seen to exhibit gradual decrease in their CA values, so that after ~300 h of immersion, the values of CA observed are within the range of ~88–95°. The observed degradation rate is somewhat different between the samples, being the fastest for sample 6. This can be explained by difference in surface chemistry and morphology (roughness), as these two parameters influence not only the surface density of self-assembled ODTMS molecules but also the strength of the Si-O-Me bond (where Me: Mn or Si) and the value of apparent CA. 

Comparison of Figure 3 and Figure 7 permits to conclude that no noticeable change was observed in surface morphology between the two-layer samples analyzed as prepared and after 72 h of immersion. In combination with the results exhibited in Figure 6, one can conclude that it was mainly the topmost ODTMS layer that degraded after interaction with water, while the under-layers remained mainly unchanged in all three cases. Since all the under-layer materials, hydrated MnO_x_, SiO_x_ or BTSE coating, are hydrophilic by nature, their exposure, upon partial removal of hydrophobic ODTMS, makes surfaces less hydrophobic and more heterogeneous. However, since all the three coatings are relatively thick, they were able to prevent water from penetrating to the underlying AA2024, which is why no signs of corrosion are observed in Figure 7. 

### 3.4. Effect of Ultrasonic Treatment

Surface images of sample 2 subjected to accelerated aging through ultrasonication in water for 1 h are shown in Figure 5c. It is clearly seen that the sample subjected to ultrasonic treatment in water demonstrates surface features similar to those in Figure 5a,b, where surface SEM images of sample 2 immersed in water for 24 h are presented. More precipitates observed in panel (Figure 5c) are most likely metal oxide/hydroxide particles redeposited on the surface after sonication, presumably formed by the material dissolved during trench formation around surface intermetallic particles. Thus, it can be concluded that the decay of surface ODTMS layer can be accelerated by sonication in water. 

The obtained results imply that ODTMS layers prepared on aluminum surfaces are not stable enough to withstand the ultrasonic rinse test for as long as 1 h. After such a test, the sample was found to demonstrate signs of surface corrosion similar to those observed after immersion in water for several tens of hours. When sonicated in water, the ODTMS–grafted surface was losing its water-repellent properties somewhat faster, while the processes involved in the coating’s degradation were likely to be essentially the same.

### 3.5. Discussion and Comparison with Previous Results

Altogether, the above results are generally consistent with previously published reports, while this study is more systematic and more focused on surfaces with different chemistry coated with ODTMS as a top-layer. Previously, the stability of several organosilane-coated metal oxide (including aluminum oxide) surfaces was tested in contact with water or ice [2,5,7,8,37]. Even though different surface treatments and silanes were applied, as well as test methods, the previous research also demonstrated that silane layers grafted on metal oxide surfaces (such as Al, Ti, W oxides) eventually decayed, either in direct contact with water or with water and ice. This typically resulted in lower CA values observed after several days of immersion, which is why in some other works, where contact time was limited to hours, such a decay was not reported. This permits to conclude that the trend observed and confirmed in this study is very general, and it is more likely to be related to the structure of organosilane molecules and the way they attach to metal oxide surface rather than to insufficient optimization of procedures used to apply such monolayers.

As an example of such a short-term contact with water, Lee et al. tested OTS layers prepared in a toluene bath on a flat Al surface [35]. At first glance, their results may appear inconsistent with what was shown above, as they observed no noticeable change in CA [35]. However, their test exploited water droplets impinging on the sample’s surface, which in total resulted in ~10–14 h of water/surface contact (as 5 L of water was consumed, with a rate being 72 drops/min, 80 µL each). Thus, the tested surfaces were not in contact with water long enough to see any significant changes in CA values of tested sample [35]. For comparison, in the present work, after full immersion in water for ~10 h, all tested samples demonstrated drop in their CA as small as just a few degrees, which is comparable with the accuracy of measurements. Similarly, Emelyanenko et al. showed that superhydrophobic pulsed-laser-roughened steel surface hydrophobized with FAS demonstrated no significant change in water CA after immersion for as long as 24 h [5]. This could be explained by: (i) different substrates (steal vs. aluminum in our case) and organosilanes used; (ii) much smaller contact area (the fraction of solid/water contact area on superhydrophobic surfaces is normally below 10%); and (iii) a much shorter time period monitored in ref. [5] (24 h vs. hundreds of hours). 

In principle, the results observed for one-layer samples 1–3 (decrease of their CA after long-term immersion in water) could be explained by not dense enough layers of ODTMS prepared on their surface. As a result, this could thus allow water molecules to penetrate through the ODTMS layer and cause electrochemical surface reactions [49,50], resulting in surface morphologies seen in Figure 5a,b. However, the fact that two-layer samples 4–6 also demonstrated similar behavior is not consistent with this assumption. As CA values on the surface of two-layer samples also decreased over time, without surface corrosion, this implies that the ODTMS top-layer decayed after reacting slowly with water. 

Thus, in accordance with previously reported assumptions [7,8,37], we conclude that water molecules can penetrate through the ODTMS layer and hydrolyze its Al-O-Si bond with the AA2024 surface (see Figure 1b,c). The bonds between alkylsilane molecules grafted to a metal oxide surface are known to form through the condensation reaction, in which the silanol group (-Si-OH) reacts with the surface metal hydroxyl group (Al-OH in case of AA2024), resulting in the formation of the metallo-siloxane bond Al-O-Si [7,26,31,32,34,36,40]:

H_3_C(CH_2_)_17_-Si-O-H + H-O-Al = H_3_C(CH_2_)_17_-O-Al + H_2_O

However, this reaction is reversible [8,34], implying that, in principle, the Al-O-Si bond can be hydrolyzed, provided that water molecules and time are available [34]. Thus, the H_3_C(CH_2_)_17_-Si-O-Al bonds may be hydrolyzed back to silanol groups, resulting in surface metal hydroxyl groups Al-OH and loose octadecylsilanol molecules H_3_C(CH_2_)_17_-Si-(OH)_3_ [34]. This is what appeared to happen gradually when the alkyl-grafted samples, both one-layer and two-layer types, were immersed in water for a long period of time. Based on Figure 4 and Figure 6, we conclude that the prepared ODTMS layers were not dense enough to protect the H_3_C(CH_2_)_17_-O-Al bonds from water molecules. Such molecules thus penetrated slowly to the ODTM-substrate interface and hydrolyzed the H_3_C(CH_2_)_17_-O-Al bond, which resulted in partial detachment of organosilane molecules and exposure of under-layer (see Figure 1b,c). 

In the case of one-layer samples 1–3, the contact of unprotected AA2024 surface with water predictably resulted in gradual electrochemical corrosion processes, which are known to proceed primarily at the intermetallic-Al matrix interface [41,42,43,44,49,50]. The results of such processes are clearly seen in Figure 5, which supports the above conclusions. In the case of samples 4–6, with two-layer coatings, degradation of their top-layer only exposed their hydrophilic under-layer, while no surface corrosion of the AA2024 substrate was observed. In both cases, however, surfaces were turning more hydrophilic and more heterogeneous over time. 

Table 2 compares chemical composition of samples 2, 4, and 5 as it was measured by XPS for both fresh (as-prepared) samples and those immersed in water for 5 days. As data for major surface elements common for all the three samples (C, O, Si, and Al) are presented, the surface content of Si atoms detected after immersion in water is seen to decrease for the one-layer sample (sample 2) and two-layer sample 4 (having under-layer based on hydrated MnO_x_). At the same time, it remains essentially unchanged for sample 5. This can be explained by the fact that the latter sample has its under-layer based on BTSE (which has its own Si atoms), which is why elimination of some surface ODTMS molecules, while partially exposing uncoated AA2024 surface in case of sample 2, uncovers Si-containing BTSE patches on sample 5. Meanwhile, after the water immersion test, the surface content of Al clearly increased only on sample 2. This is quite expected, as the two-layer samples 4 and 5 remained covered by their under-layer (MnO_x_ or BTSE) even after partially losing their ODTMS top-layer. In case of sample 4, the increase in surface oxygen content observed after immersion is also consistent with a partial loss of ODTMS layer and disclosure of MnO_x_ under-layer. Thus, the XPS results presented in Table 2 are consistent with our conclusions. 

Note that the stability of the anticorrosive coating on metal surfaces can also be checked by taking SEM images of immersed samples over time [54]. However, the surface morphology of polished AA2024 coated with ODTMS is very inhomogeneous because of second-phase particles with different morphology and chemical composition. That is why following the degradation of the ODTMS layer over such a surface would be very difficult, and following changes in CA over time as the thin ODTMS layer decays is much more practical. 

## 4. Concluding Remarks

This work systematically studied how the hydrophobicity of alkylsilane layers formed on aluminum surfaces from octadecyltrimethoxysilane (ODTMS) changed over time after immersion in water. The samples were prepared via immersing polished aluminum alloy (AA2024) plates in an ODTMS bath at its natural pH (~5.2) with further coating polycondensation caused by aging at 50 °C in air. Two series of samples were prepared, being one-layer and two-layer coatings, with single ODTMS or ODTMS coatings on an under-layer formed by hydrated MnO_x_, BTSE, or a hydrated SiO_x_ layer. 

To study their long-term stability in contact with water, all the samples were immersed in deionized water, and their water contact angle was measured systematically for as long as several weeks (typically at least 2–4). For comparison, another one-layer ODTMS sample was subjected to the ultrasonic rinse test, which simulated a long-term water immersion test over a short period of time. The highest stability over time was demonstrated by a one-layer sample prepared in ethanol/water bath for 5 min and by a similar ODTMS layer prepared on hydrated MnO_x_ as an under-layer.

As all the samples exhibited a decrease in their hydrophobicity over time, it is concluded that ODTMS layers get hydrolyzed gradually and slowly degrade, letting water molecules interact with their underlying layers. This was also confirmed by both SEM and XPS investigations. Since ODTMS coatings prepared on Al alloy via dip-coating proved to be unstable, for comparison, their counterpart layers prepared via CVD should be tested in the future.

## Figures and Tables

**Table 1:** Samples and details of their preparation.

Sample	Under-Layer	ODTMS Bath Immersion Time (min)	Bath Composition (*v*/*v* Ratio)	Initial Water CA Value (°)
1	-	0.5	9:1 (EtOH/H_2_O)	104.4
2	-	5	9:1 (EtOH/H_2_O)	105.1
3	-	5	9:1 (MeOH/H_2_O)	102.1
4	PCC	5	9:1 (EtOH/H_2_O)	106.2
5	BTSE	5	9:1 (EtOH/H_2_O)	104.4
6	SiO_x_	5	9:1 (EtOH/H_2_O)	110.8

**Table 2:** Elemental composition of samples 2 and 5 as measured by XPS before and after their immersion in water for 120 h.

Sample	Detection Time	Element (at. %)			
		C	O	Si	Al
2	Before immersion	43.2	31.3	13.1	12.4
	After immersion	27.5	38.7	4.7	29.1
4	Before immersion	46.7	33.6	19.7	0
	After immersion	47.9	39.7	12.4	0
5	Before immersion	47.3	29.8	22.9	0
	After immersion	48.8	27.7	21.8	1.7

## Data Availability

Not applicable.

## Supplementary Materials

The following are available online at https://www.mdpi.com/article/10.3390/ma15051804/s1, Table S1: Elemental composition of alloy AA2024, Table S2: Surface roughness of samples 1–3 evaluated by surface profiler.
