# Supplementary material for: Stability of Octadecyltrimethoxysilane-Based Coatings on Aluminum Alloy Surface
Source: Materials (Basel). 2022 Feb 28;15(5):1804. doi: 10.3390/ma15051804 (PMC8911556; doi:10.3390/ma15051804)
Supplement: Supplementary file 1 [file materials-15-01804-s001.zip › materials-1539437-supplementary.pdf]

Supplementary Materials:

# Stability of Octadecyltrimethoxysilane- Based Coatings on Aluminum Alloy Surface

Alexey Y. Zhizhchenko [1,2], Anastasiia V. Shabalina [3], Ali A. Aljulaih [4,5], Stanislav O. Gurbatov [1,2], Aleksandr A. Kuchmizhak [1,2], Satoru Iwamori [4] and Sergei A. Kulinich [1,4,*]

**Table S1.** Elemental composition of alloy AA24.(from: ASM Aerospace Specifications Metals Inc.).

| Element | Cu | Mg | Fe | Mn | Si | Cr | Zn | Ti | Others | Al |
|---|---|---|---|---|---|---|---|---|---|---|
| Weight % | 3.8- 4.9 | 1.2- 1.8 | < 0.5 | 0.3- 0.9 | < 0.5 | < 0.1 | < 0.25 | < 0.15 | < 0.15 | 90.7 – 94.7 |

**Table S2**. Surface roughness of samples 1-3 evaluated by surface profiler

| Sample | 1 | 2 | 3 |
|---|---|---|---|
| $R_a$ (nm) | 310 ±15 | 351 ±19 | 342 ±20 |
| RMS (nm) | 321 ±21 | 355 ±25 | 349 ±28 |
